# Risk Evaluation of Bone Metastases and a Simple Tool for Detecting Bone Metastases in Prostate Cancer: A Population-Based Study

**DOI:** 10.1155/2023/9161763

**Published:** 2023-02-14

**Authors:** Shi Li, Caixia Chen, Hua Zhu, Qi Lin, Zhixian Yu

**Affiliations:** ^1^Department of Urology, The First Affiliated Hospital of Wenzhou Medical University, Wenzhou, 325000 Zhejiang, China; ^2^Department of Clinical Laboratory, Key Laboratory of Clinical Laboratory Diagnosis and Translational Research of Zhejiang Province, The First Affiliated Hospital of Wenzhou Medical University, Wenzhou, 325000 Zhejiang, China; ^3^Haikou Branch of Hainan Food and Drug Inspection Institute, Haikou, 570311 Hainan, China; ^4^Department of Obstetrics and Gynecology, The First Affiliated Hospital of Wenzhou Medical University, Wenzhou, 325000 Zhejiang, China

## Abstract

**Introduction:**

Population-based estimates of the incidence and prognosis of bone metastases in prostate cancer (PC) are lacking. We aimed to characterize the incidence and risk of bone metastases and develop a simple tool for the prediction of bone metastases among patients with PC.

**Methods:**

Data were obtained from the Surveillance, Epidemiology, and End Results (SEER) database. A total of 75698 patients with PC with confirmed presence or absence of bone metastases at diagnosis between 1975 and 2019 in the United States were used for analysis. Data were stratified by age, race, residence, median income, prostate-specific antigen (PSA) values, tumor size, distant metastatic history, and positive lymph node scores. Multivariable logistic and Cox regressions were performed to identify predictors of bone metastases and factors correlated with all-cause mortality. Classification tree analysis was performed to establish a model.

**Results:**

After patients with PC with missing data were excluded, 75698 cases remained. Among these, 3835 patients had bone metastases. Incidence proportions were highest in patients with a high prostate-specific antigen (PSA) value (odds ratio (OR), 2.49; 95% confidence interval (CI), 1.35-4.35; *p* < 0.002). Multivariable Cox regression and risk analyses indicated that high PSA values (hazards ratio (HR), 19.8; 95% CI, 18.5-21.2; *p* < 0.001) and high positive lymph node scores (vs. score 0; HR, 8.65; 95% CI, 7.89-9.49; *p* < 0.001) were significant risk factors for mortality. Meanwhile, in the predication tree analysis, PSA values and lymph node scores were the most significant determining factors in two models. Median survival among the patients with PC was 78 months, but only 31 months among those with bone metastases.

**Conclusion:**

Patients with PC with high PSA values or high positive lymph node scores were at a significantly higher risk of bone metastases. Our study may provide a simple and accurate tool to identify patients with PC at high risk of bone metastases based on population-based estimates.

## 1. Introduction

Prostate cancer (PC) is one of the most common malignant diseases globally; it was the fourth most commonly diagnosed cancer in both sexes and the second most frequently occurring cancer in males in 2020 [1]. With the development of new treatment technology, especially the robot-assisted surgery system, the survival of patients with PC has increased dramatically in recent years. But the prevalence of distant metastasis has also increased during this process [2]. Bone is the most frequent distant metastasis site of PC. Bone metastases can result in bone destruction and lead to considerable disability and impaired quality of life [3]. Furthermore, patients with PC with bone metastases have a much poorer prognosis and notably higher mortality than those without bone metastases. Only 3% of patients with bone metastases survive after five years [4]. Therefore, detecting bone metastasis or accurately evaluating its risk in early PC diagnosis is critical.

At present, urologists assess the risk of bone metastases based on their own clinical experience or single diagnostic examination result, such as positron emission tomography/computed tomography (PET/CT) [5]. Some surgeons have also tried to establish a tool to help them assess the risk of bone metastases, but most of these studies are based on a single institution or their own experiences [6, 7]. Large population-based studies that focus on the risk assessment of bone metastases in PC are deficient. Furthermore, no study has been published suggesting a simple tool specifically for the risk assessment of bone metastases in patients with PC. Therefore, it is important to build an easy and reliable predictive tool. With the development of computer science, machine learning has become widely used in medical and healthcare applications, especially in the development of new diagnostic or prediction systems [8]. This phenomenon accelerated and became more widely used after the breakout of the coronavirus disease 2019 pandemic (COVID-19) [9]. The Surveillance, Epidemiology, and End Results (SEER) database provides detailed information on cancer statistics for the United States (US) population [10]. SEER is widely used for deep matching learning and seeking new diagnostic methods [11].

The objective of this study was to assess the basic clinical characteristics in patients with PC with or without bone metastases. Furthermore, we aimed to evaluate the survival details and investigate both clinical and sociodemographic predictors of bone metastasis among patients with PC. Finally, we used these data to develop and evaluate a simple tool to predict the risk of bone metastases that can be used in daily clinical work.

## 2. Methods

### 2.1. Database

The SEER database includes cancer incidence data from large-scale population-based cancer registries covering approximately 47.9% of the US population from 1975 to 2019. Information on the presence or absence of distant metastases at the time of first PC diagnosis was released from January 1, 2010, to December 31, 2015. In addition, the database collects patient demographics such as age, race, and sex, as well as clinical information like primary tumor site, tumor morphology, stage at diagnosis, first course of treatment, and follow-ups with patients for vital status. We identified 90812 cases of patients who were diagnosed as having PC with or without bone metastases at the first step.

### 2.2. Study Population

**Figure 1:**
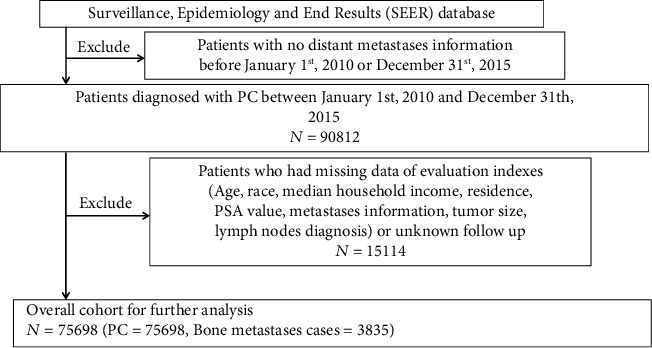
Flow diagram showing the selection of patients from the Surveillance, Epidemiology, and End Results (SEER) database.

We recruited patients diagnosed with PC (International Classification of Diseases- (ICD-) 0-3/World Health Organization (WHO) 2008: “Prostate”; histology recode: 8140-8339) between January 1, 2010, and December 31, 2015 (Figure 1). Then, we chose to include the age at diagnosis, race, median household income, residence, prostate-specific antigen (PSA) value, metastases diagnosis, tumor size, lymph node diagnosis, survival follow-up, and death classification for evaluation in our study. Among these patients with PC, we excluded 15114 cases where important clinical or basic information was unknown or missing. After exclusion, 75698 cases were included in the final cohort for further analysis.

### 2.3. Definition of Incidence

Patients were stratified by their PSA values: PSA < 10 was defined as the low-risk group, 10≦PSA < 10-20 was defined as the intermediate risk group, and PSA ≥ 20 was defined as the high risk group. Incidence proportion was also assessed among patients with positive lymph node scores or metastases to distant sites. Absolute numbers and incidence proportions were calculated for patients with PC with bone metastases or other distant metastases at initial diagnosis. Age, race, household income, and place of residence were also evaluated to determine whether baseline characteristics can affect these factors. The baseline or clinical information was categorized according to the SEER database.

### 2.4. Statistical Analysis

Based on the SEER database, age was categorized as “≤60,” “61-70,” “71-80,” and “≥81.” Race was divided into five groups: “White,” “Black,” “American Indian/Alaska Native,” “Asian or Pacific Islander,” and “unknown.” Household income (USD) was classified as “low (≤59999),” “median (60000-74999),” and “high (≥75000).” The place of residence was grouped as “urban” and “rural” according to the SEER database. Median household income and residence were linked at the county level, and the same value was used regardless of diagnosis/death years, according to the SEER database. The number of metastases was categorized as “0,” “1,” and “≥2.” Tumor size was categorized as “small (<999)” and “large (≥999).” Finally, the positive lymph node score was categorized as “0,” “100,” “800,” and “999” using the original classification method in the database.

We used multivariable logistic regression to determine these baseline indexes, including age, race, residence type, and household income, between patients with PC and PC cases with bone metastases. In addition, relevant disease information, including PSA value, tumor size, lymph node index, and the presence of bone, lung, liver, or brain metastases, was available in the SEER database. This disease information was used to characterize the extent of systemic disease. For multivariable logistic regression analyses, we used the “Stas19” package and “glm” function in R software (version 4.2.0.; R Core Team, Vienna, Austria).

The Kaplan-Meier method was used for survival analyses and drawing the survival curve. In addition, multivariable Cox regression was carried out to evaluate the hazard ratio (HR) in different subgroups among patients with PC, with or without bone metastases. This analysis helped us to identify variates correlated with increased all-cause mortality. The “ezcox” and “survival” packages in R software were used to perform these analyses.

We also built a model via the classification tree method using the “rpart” package of R software. Tree classification is based on nonlinear discrimination analyses. First, it splits a data sample into different subgroups using independent variables. Then, the dependent factors most strongly associated with the independent variable were identified using R software. Bone metastasis was the independent variable, and clinical or sociodemographic characteristics were the dependent variables. To guarantee the reliability of the tree model, we devised two different methods to detect the mean decrease Gini (MDG). To further confirm our conclusion, we randomly collected 300 cases from the data as a subgroup. For this subgroup, we carried out a decision tree classification model using the software package SPSS 18.0 (SPSS Inc., Chicago, IL, USA).

## 3. Results

### 3.1. Number and Incidence Proportions of Patients with PC in Different PSA Groups

The number and incidence proportions of PC cases with or without bone metastases were stratified by PSA values (Table 1). Among the 75698 patients with PC, 58183 (76.86%) had low PSA values, 12487 (16.5%) had medium values, and 10028 (13.25%) had high values. Of all the patients with PC, there were 3835 cases of bone metastases. Among these patients, 350 (9.13%) had low PSA values, 356 (9.28) had medium, and 3129 (81.60%) had high PSA values. Among the 3920 patients with PC with distant metastases, 366 (9.34%) had low PSA values, 366 (9.34%) had medium PSA values, and 3188 (81.33%) had high PSA values. Incidence proportions were highest among cases with high PSA values (31.20% of the entire cohort and 98.15% of the metastatic subgroup) and lowest among patients with low PSA values (0.60% of the entire cohort and 95.63% of the metastatic subgroup). In the group with medium PSA values, 356 patients presented with bone metastases, accounting for 2.85% of the entire study cohort and 97.27% of the metastatic subgroup.

### 3.2. Multivariable Logistic Regression for Bone Metastases in Patients with PC

A high PSA value (vs. a low PSA value; odds ratio (OR), 2.49; 95% confidence interval (CI), 1.35-4.35; *p* < 0.002) was significantly different, but a medium PSA value (vs. a lower PSA; OR, 1.67; 95% CI, 0.75-3.87; *p* = 0.214) was not. Living in rural areas, a lower household income, a larger tumor size, and a higher positive lymph node score were positively correlated with the incidence of bone metastases; however, these factors were not significant. Neither age at diagnosis nor race was significantly associated with an increased risk of bone metastases in PC cases. These results are presented in Table 2.

### 3.3. Survival Analyses in Patients with PC with Bone Metastases

**Figure 2:**
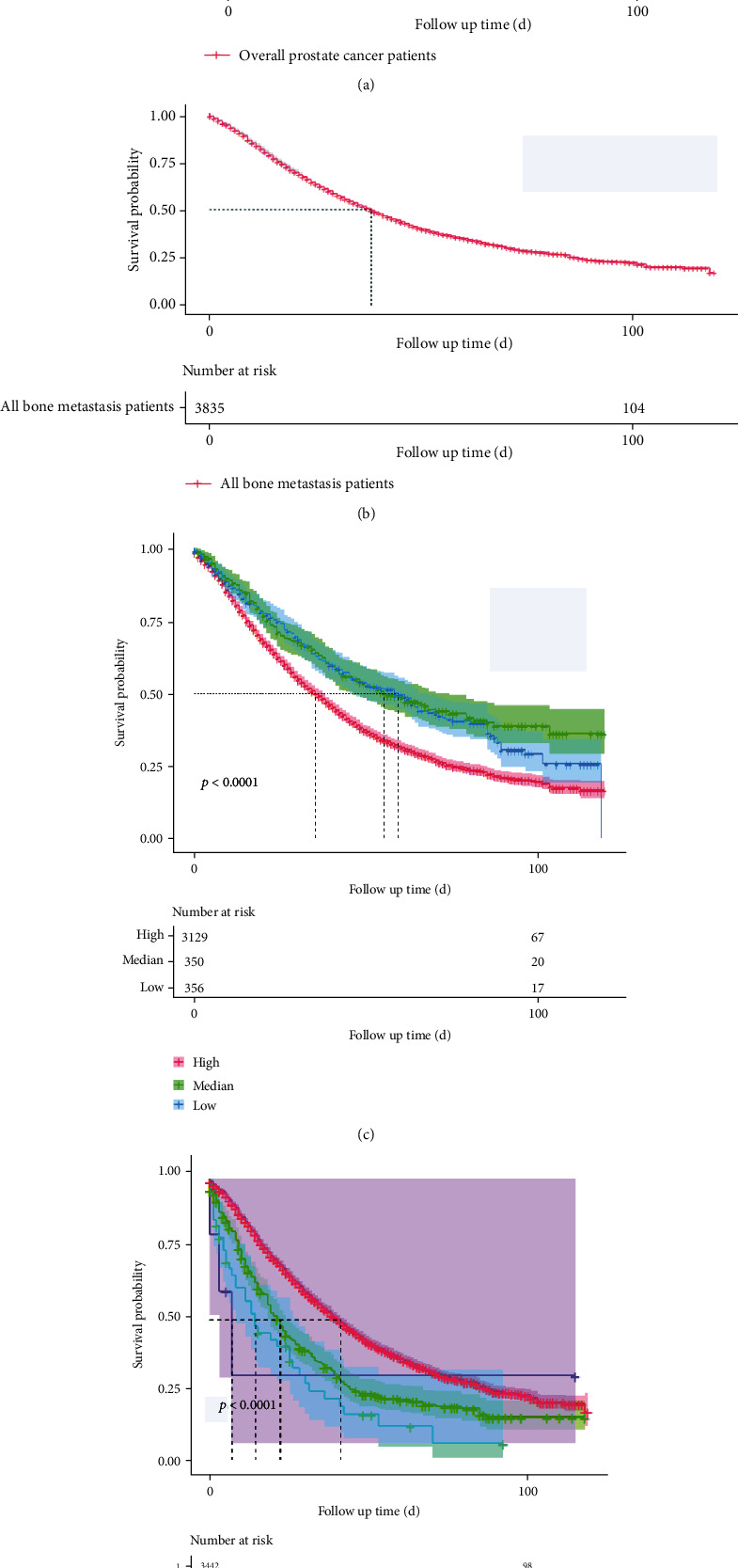
Overall survival among patients with PC and patients with PC with bone metastases: (a) overall survival among patients with PC; (b) overall survival among patients with PC with bone metastases; (c) survival in bone metastasis cases stratified by different PSA values; (d) survival in patients with PC with bone metastases stratified by the number of distant metastatic organs.

As stratified by PSA values, the median survival times among patients with PC (*n* = 75698) and patients with bone metastases (*n* = 3835) are shown in Table 1. In both the total PC cases and the bone metastases subgroup, patients with low PSA values had a longer median survival compared with patients with higher PSA values. Furthermore, survival in the overall patients with PC cohort (Figure 2(a)) was much longer than that of the metastasis cases (Figure 2(b)). In bone metastasis cases, overall survival estimates (Figure 2(b)) and those stratified by different PSA values (Figure 2(c)) and by the number of distant metastatic organs (Figure 2(d)) are also graphically displayed. Patients with PC with lower PSA values or fewer metastases had longer survival times.

### 3.4. Multivariable Cox Regression and Risk Analyses for the Bone Metastasis Incidence in Patients with PC

Table 3 shows the results of multivariable Cox regression for all-cause mortality among patients with PC with bone metastases.

In all patients with PC, the risk of all-cause mortality was significantly higher in the following categories:

1. Patients aged 60-70 years (vs. age 13-60; HR, 1.18; 95% CI, 1.09-1.28; *p* < 0.001), aged 70-80 years (vs. age 13-60; HR, 2.34; 95% CI, 2.16-2.55; *p* < 0.001), and aged >80 years (vs. age 13-60; HR, 9.85; 95% CI, 9.01-10.8; *p* < 0.001)
2. Patients living in rural districts (vs. urban districts; HR, 1.31; 95% CI, 1.22-1.41; *p* < 0.001) and patients with medium PSA values (vs. low PSA values; HR, 3.18; 95% CI, 2.91-3.48; *p* < 0.001) and high PSA values (vs. low PSA values; HR, 19.8; 95% CI, 18.5-21.2; *p* < 0.001)

For distant metastases, the risk of all-cause mortality was significantly higher in the following categories: (1) patients with one metastasis (vs. no metastasis; HR, 37.4; 95% CI, 35.2-39.6; *p* < 0.001) and multimetastases (vs. no metastasis; HR, 66.2; 95% CI, 58.7-74.6; *p* < 0.001), (2) large tumor size (vs. small size; HR, 2.55; 95% CI, 2.27-2.86; *p* < 0.001), and (3) lymph node score 100 (vs. score 0; HR, 10.5; 95% CI, 9.76-11.2; *p* < 0.001), lymph node score 800 (vs. score 0; HR, 20.8; 95% CI, 16.2-26.6; *p* < 0.001), and lymph node score 999 (vs. score 0; HR, 8.65; 95% CI, 7.89-9.49; *p* < 0.001).

Neither race nor income was significantly associated with elevated mortality for all patients with PC.

In patients with PC with bone metastases, the risk of all-cause mortality was significantly higher in the following categories:

1. Patients with a high PSA value (vs. low PSA; HR, 1.6; 95% CI, 1.37-1.86; *p* < 0.001)
2. Multimetastases (vs. one metastasis; HR, 1.74; 95% CI, 1.54-1.96; *p* < 0.001)
3. Lymph node score 100 (vs. score 0; HR, 1.28; 95% CI, 1.16-1.4; *p* < 0.001), lymph node score 800 (vs. score 0; HR, 1.39; 95% CI, 1.06-1.84; *p* = 0.0188), and lymph node score 999 (vs. score 0; HR, 1.32; CI, 1.18-1.47; *p* < 0.001)

Considering that the number of cases with bone metastases was limited, no significance was found between mortality and factors such as age, race, residence district, income, or tumor size.

**Figure 3:**
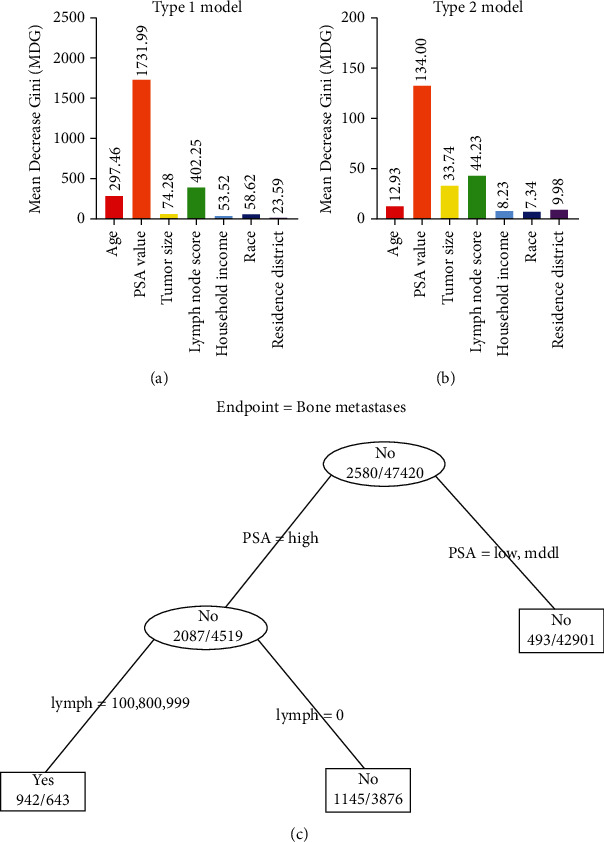
Mean decrease Gini (MDG) expression in two different models using random forest algorithm and prediction tree model for bone metastases. In either the (a) type 1 model or (b) the type 2 model, the PSA value had the largest MDG, followed by positive lymph node scores. (c) Classification tree for predicting bone metastases among patients with PC.

In general, poorer survival prognosis was correlated with a series of clinical and sociodemographic factors, especially PSA values, metastases, and positive lymph node scores. Furthermore, we applied these clinical and sociodemographic characteristics for deeper analyses using the random forest method and decision trees to explore the association between these factors and bone metastasis risk. Using two different models in the random forest algorithm, we observed that the PSA value had the largest MDG. The MDG expression was 1731.99 in the type 1 model (Figure 3(a)) and 134.00 in the type 2 model (Figure 3(b)). Following the PSA value, the lymph node score also had a relatively higher MDG expression than the other factors in both models. These results indicated that the PSA value and lymph node score at diagnosis had the greatest association with the incidence of bone metastasis in patients with PC.

The decision tree illustrates that among all the clinical and nonclinical parameters in our analyses, high PSA values and positive lymph node scores could be good indexes for predicting bone metastases in patients with PC (Figure 3(c)). High PSA was the most important determining factor, which was the first-level split of two initial branches of the tree. The accuracy of the tree is 95.90%. To further guarantee accuracy, 300 cases were randomly collected as a subtype group from our data. Decision tree classification was carried out on this 300-case subgroup, and it reconfirmed that a higher PSA value was a critical factor indicating bone metastases (Supplementary Figure 1A, B).

## 4. Discussion

The number of patients with PC is increasing worldwide, and the prognosis of patients with PC varies according to factors such as age and race [12]. PC is prone to distant metastasis, leading to a very poor prognosis. Among these metastases, bone metastasis accounts for the largest proportion [3]. More than half of patients already have bone metastases at their initial diagnosis [13].

In this study, we described the incidence of identified bone metastases among patients with newly diagnosed PC and evaluated the patients' risk and survival characteristics based on the SEER database, a large population-based database. Due to the fact that the SEER database only provided bone metastasis information at the initially diagnosis, its incidence is likely to have been underestimated. However, large data still ensures the reliability and accuracy of our results. A higher PSA value was significantly correlated with a higher incidence of bone metastases and indicated a poorer survival prognosis. Median survival time ranged from 57 months in patients with high PSA values to 82 months in patients with low PSA values. Based on these results, a tree model was established to assess the bone metastasis risk, which could help doctors identify those at a high risk.

Bone is one of the most common organs affected by metastases in human cancers, especially in lung cancer, PC, and breast cancer [14]. Compared with patients with PC without bone metastasis, cases with bone metastasis have significantly shorter survival (2.2 years vs. 3.5 years) and higher mortality (73% vs. 19%). Bone metastases serve as a major cause of death in patients with PC [15]. Furthermore, most cases of bone metastasis are also castration-resistant. Therefore, there are no effective treatments for these cases [16].

The PSA test is a risk assessment tool for PC diagnosis and prognosis evaluation [17]. Some recent studies have used the nadir PSA level and time to nadir for prognosis assessment in castration-resistant patients with PC [18]. In most of these studies, incidence and survival results were not stratified by PSA values or other subtypes. Some urologists also use advanced medical examination such PET-CT or bone scintigraphy to evaluate the risk of bone metastases in patients with PC [19]. Most of these examinations are expensive, and patients are often not willing to take these tests in the initial stages of their disease. Therefore, developing a simple and easy tool is important for patients with PC, especially for financially disadvantaged patients. With the development of artificial intelligence technology, machine learning can help us to develop new and easy diagnostic or predictive tools in clinical work, based on population-based big data. It has been applied in many common diseases, such as tuberculosis [20] and Alzheimer's disease [21]. Furthermore, some physician scientists use machine deep learning to pioneer the next generation of medical robotics [22].

A weakness of our study is that information about bone metastases was available at first presentation rather than at any time over the disease course. But the SEER database provides a population-based big data information in a long time from 1975 to 2019. It can ensure accuracy and reliability of our conclusion in real work. In our study, we focused on the association between the risk of bone metastases and PSA values as well as other baseline or clinical characteristics. We observed that 5.07% of patients with PC with bone metastases at initial diagnosis and 97.8% of cases with metastatic PC cancer at any distant site had bone metastases. Among the entire cohort, we found that PC cases with high PSA values (vs. low PSA cases) had significantly higher odds of suffering bone metastases at initial diagnosis. Furthermore, our data also indicated that patients with PC who lived in rural areas and had lower household income, larger tumor sizes, or higher positive lymph node scores were positively correlated with the incidence of bone metastases; however, these factors were not statistically significant. Many studies have indicated that clinical and socioeconomic factors may affect survival among patients with malignancies [23, 24]. In our study, the median survival for patients with PC was 78 months and only 31 months among patients with bone metastases. Our data indicated that the HR varied by subtypes in all patients with PC and in those with bone metastases. Patients with PC with high PSA values had the highest HR value (vs. low PSA: HR, 19.8; 95% CI, 18.5-21.2; *p* < 0.001 in all PC cases and in bone metastasis cases, vs. low PSA: HR, 1.6; 95% CI, 1.37-1.86; *p* < 0.001). Patients with high PSA values had the worst survival in bone metastasis cases. Besides the PSA value, we also found that a higher positive lymph node score was positively correlated with higher HR values. Our results are consistent with some general trends reported in previous studies [25, 26].

Meanwhile, other baseline characteristics, including age, income, tumor size, and residence area, also affected the HR values. We constructed a decision tree model based on the HR and conducted survival analyses for bone metastasis prediction in patients with newly diagnosed PC. In this model, we found that patients with high PSA scores and positive lymph nodes required close attention to the possibility of bone metastases during their follow-up medical evaluations. In comparison to other evaluation methods in clinical work, the prediction tree model is the most convenient for urologists and researchers. The tree is easily processed from the root to the terminal nodes through several median branches. This process depends on the simple choice question of “yes” or “no.” It is much easier for clinical workers to analyze daily clinical information or samples from big public databases compared to other complex mathematical methods [27].

## 5. Strengths and Limitations

The strengths of our study are that our sample was large and included diverse population-based cases and detailed clinical information obtained directly from surgeons and physicians which guarantees the research's reliability. Meanwhile, the predication tool is simple and reliable for urologists to assess the risks of bone metastases of their patients with PC quickly and noninvasively.

However, several limitations should be considered. The primary limitation is that most of the information on these patients with PC that were provided by the SEER database was obtained during the first treatment in the hospital; hence, patients who subsequently developed bone metastasis later in the course of their disease could not be included in our evaluation. This is an important limitation that should be considered by other researchers in the future. Also, the median residence type and median household income in SEER were defined at a county level, not a personal level. To a certain extent, this may affect our analyses. Furthermore, we have no data on other important characteristics such as patients' smoking status, alcohol use, and reasons why patients and their doctors chose a particular treatment. Finally, the SEER database does not provide information on the treatment that these patients with PC received.

## 6. Conclusions

Considering these limitations, we will consider building our own PC clinical database. Our database will include some valuable treatment information and follow-up data after surgery, as well as the basic factors provided by the SEER database. We believe it will help urologists to evaluate the prognosis, including the risk of bone metastases after surgery treatment. It could be a meaningful addition to the simple tool we developed in this study. Big public databases can guarantee the reliability of this study; however, it may lack some valuable clinical information. Finally, we will integrate our own database with the big public database to carry out further analysis and future study.

Our research explores the epidemiology of bone metastases in patients with PC in the US. Patients with PC with high PSA values, lower household income, larger tumor size, and high positive lymph node scores are more likely to have poor prognosis and survival. Moreover, patients with high PSA values are at a significantly higher risk of bone metastases. In addition, high positive lymph node scores also indicate a high risk of bone metastases among patients with PC. A simple tree model for prediction of bone metastases among patients with PC has been created based on these findings. Our research provides useful information for urologic clinical research, as well as an easy and simple diagnostic tool for urologists and patients with PC in clinical diagnosis and treatment.

## Figures and Tables

**Table 1:** Incidence proportion and median survival of patients with PC or patients with PC with bone metastases by subtype (PSA values at diagnosis).

Subtype (PSA)	Patients (no.)			Incidence proportion of bone metastases (%)		Survival among PC patients, median, mo	Survival among PC patients with bone metastases, median, mo
	With PC	With metastatic disease	With bone metastases	Among entire PC patients	Among subset with metastatic disease		
Low	53183	366	350	0.66	95.63	82	43
Median	12487	366	356	2.85	97.27	74	44
High	10028	3188	3129	31.2	98.15	57	28

**Table 2:** Multivariable logistic regression for the presence of bone metastases among patients with PC.

Variable	Patients (*N*)	With metastatic diseases (*n* = 3920)	With bone metastases (*n* = 3835)	Among entire cohort	
	Patients (*n* = 75698)			OR (95% CI)	*p* value
*Age at diagnosis (years)*					
≤60	21297	704	695	Reference	NA
61-70	33328	1254	1224	0.55 (0.24-1.13)	0.12
71-80	17030	1062	1039	0.59 (0.26-1.26)	0.19
≥81	4043	900	877	0.46 (0.20-1.00)	0.06
*Race*					
White	59841	2988	2922	Reference	NA
Asian or Pacific Islander	5022	313	308	1.50 (0.64-4.39)	0.40
Black	9471	562	549	0.83 (0.46-1.63)	0.57
American Indian/Alaska Native	432	46	45	0.84 (0.17-15.11)	0.86
Unknown	932	11	11	NA	NA
*Residence*					
Urban	65580	3291	3216	Reference	NA
Rural	10118	629	619	1.53 (0.73-3.44)	0.28
*PSA value*					
Low	53183	366	350	Reference	NA
Median	12487	366	356	1.67 (0.75-3.87)	0.21
High	10028	3188	3129	2.49 (1.35-4.35)	0.002
*Median household income*					
High	30681	1523	1486	Reference	NA
Median	27533	1287	1262	1.32 (0.78-2.25)	0.30
Low	17484	1110	1087	1.06 (0.59-1.97)	0.85
*Tumor size*					
Small	10542	178	173	Reference	NA
Large	65156	3742	3663	1.25 (0.43-2.87)	0.63
*Lymph node score*					
0	71029	2202	2151	Reference	NA
100	2822	989	971	1.20 (0.70-2.15)	0.52
800	106	71	70	1.62 (0.34-28.93)	0.64
999	1741	658	643	0.93 (0.53-1.74)	0.81
					

**Table 3:** Multivariable Cox regression for all-cause mortality among prostate cancer patients and bone metastasis cases.

Variable	Patients (*N*)	With metastatic diseases (*n* = 3920)	With bone metastases (*n* = 3835)	All-cause mortality among overall cases		Bone metastasis specimens' mortality	
	Patients (*n* = 75698)			Hazard ratio (95% CI)	*p* value	Hazard ratio (95% CI)	*p* value
*Age at diagnosis (years)*							
≤60	21297	704	695	1 (reference)	NA	1 (reference)	NA
61-70	33328	1254	1224	1.18 (1.09-1.28)	<0.001	0.93 (0.83-1.05)	0.26
71-80	17030	1062	1039	2.34 (2.16-2.55)	<0.001	1.13 (1.00-1.27)	0.05
≥81	4043	900	877	9.85 (9.01-10.8)	<0.001	1.65 (1.46-1.87)	<0.001
*Race*							
White	59841	2988	2922	1 (reference)	NA	1 (reference)	NA
Asian or Pacific Islander	5022	313	308	0.98 (0.87-1.1)	0.68	0.73 (0.62-0.86)	<0.001
Black	9471	562	549	0.15 (0.05-0.29)	1.26	1.06 (0.95-1.19)	0.29
American Indian/Alaska Native	432	46	45	2.19 (1.69-2.84)	<0.001	1.18 (0.82-1.69)	0.37
Unknown	932	11	11	0.16 (0.09-0.31)	<0.001	0.48 (0.18-1.29)	0.15
*Residence*							
Urban	65580	3291	3216	1 (reference)	NA	1 (reference)	NA
Rural	10118	629	619	1.31 (1.22-1.41)	<0.001	1.04 (0.93-1.16)	0.5
*PSA value*							
Low	53183	366	350	1 (reference)	NA	1 (reference)	NA
Median	12487	366	356	3.18 (2.91-3.48)	<0.001	1.08 (0.88-1.32)	0.48
High	10028	3188	3129	19.8 (18.5-21.2)	<0.001	1.60 (1.37-1.86)	<0.001
*Median household income*							
High	30681	1523	1486	1 (reference)	NA	1 (reference)	NA
Median	27533	1287	1262	1.05 (0.99-1.12)	0.13	1.06 (0.97-1.17)	0.21
Low	17484	1110	1087	1.34 (1.25-1.44)	<0.001	1.07 (0.97-1.19)	0.15
*Distant metastatic sites to the bone, lung, brain, or liver*							
0	71778	0	0	1 (reference)	NA	NA	NA
1	3514	3514	3442	37.4 (35.2-39.6)	<0.001	1 (reference)	NA
Multi	406	406	393	66.2 (58.7-74.6)	<0.001	1.74 (1.54-1.96)	<0.001
*Tumor size*							
Small	10542	178	173	1 (reference)	NA	1 (reference)	NA
Large	65156	3742	3663	2.55 (2.27-2.86)	<0.001	1.12 (0.92-1.37)	0.28
*Lymph node score*							
0	71029	2202	2151	1 (reference)	NA	1 (reference)	NA
100	2822	989	971	10.5 (9.76-11.2)	<0.001	1.28 (1.16-1.4)	<0.001
800	106	71	70	20.8 (16.2-26.6)	<0.001	1.39 (1.06-1.84)	0.02
999	1741	658	643	8.65 (7.89-9.49)	<0.001	1.32 (1.18-1.47)	<0.001

## Data Availability

Data analyzed in the current study are available from the corresponding authors on reasonable request.

## Supplementary Materials

Supplementary Materials To further confirm our conclusion, we randomly collected 300 cases from the data as a subgroup. For this subgroup, we carried out a decision tree classification model using the software package SPSS 18.0. The result reconfirmed that a higher PSA value was a critical factor indicating bone metastases.
