# Supplementary material for: Risk Evaluation of Bone Metastases and a Simple Tool for Detecting Bone Metastases in Prostate Cancer: A Population-Based Study
Source: Comput Math Methods Med. 2023 Feb 14;2023:9161763. doi: 10.1155/2023/9161763 (PMC9943600; doi:10.1155/2023/9161763)

A

Model Summary

Specifications	Growing Method	CHAID
	Dependent Variable	BoneMeta
	Independent Variables	Race, PSA, size, lymph, Age, residence, income
	Validation	None
	Maximum Tree Depth	3
	Minimum Cases in Parent Node	100
	Minimum Cases in Child Node	50
Results	Independent Variables Included	PSA
	Number of Nodes	3
	Number of Terminal Nodes	2
	Depth	1

B

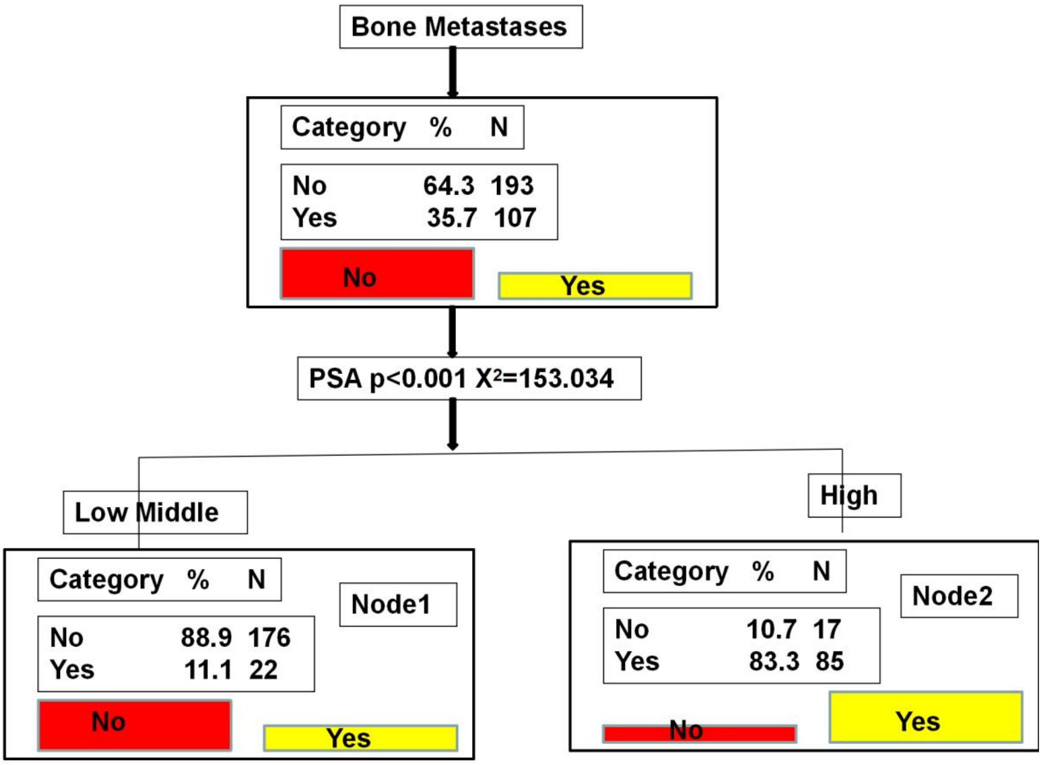

Supplement: Supplementary Materials — To further confirm our conclusion, we randomly collected 300 cases from the data as a subgroup. For this subgroup, we carried out a decision tree classification model using the software package SPSS 18.0. The result reconfirmed that a higher PSA value was a critical factor indicating bone metastases. [file 9161763.f1.pdf]
